# Troponina – Use com Sabedoria. E como mais um Instrumento na Clínica

**DOI:** 10.36660/abc.20220353

**Published:** 2022-06-06

**Authors:** Ricardo Wang, Estevāo Lanna Figueiredo

**Affiliations:** 1 Universidade Federal e Minhas Gerais Instituto Orizonti Hospital das Clínicas Belo Horizonte MG Brasil Instituto Orizonti Hospital das Clínicas da Universidade Federal e Minhas Gerais, Belo Horizonte, MG – Brasil

**Keywords:** Infarto do Miocárdio, Biomarcadores, Troponina, Mortalidade, Epidemiologia, Isquemia Miocárdica, Revascularização Miocárdica, Síndrome Coronariana Aguda, Prognóstico

O infarto agudo do miocárdio (IAM) junto com a doença coronariana crônica estável são as principais causas de mortalidade no Brasil.^1^ Em 2019 foi responsável por mais de 170.000 óbitos no Brasil. Dado à sua gravidade, houve, na Cardiologia, grande empenho na melhoria constante das ferramentas para o diagnóstico correto, na tentativa de evitar a liberação de pacientes com Síndrome Coronariana Aguda (SCA), e suas consequências clínicas e jurídicas. São considerados pilares para o diagnóstico, além de uma boa anamnese com a caracterização do tipo da dor, as alterações eletrocardiográficas e os biomarcadores (principalmente a troponina).

Os biomarcadores têm um papel importante no reconhecimento da SCA, e os algorítmicos de diagnóstico foram se adaptando à medida que evoluíam. No início, eram marcadores inespecíficos (p.e.: desidrogenase lática, transaminase oxacética, creatinofosfoquinase total – CK). Depois evoluiram para um marcador um pouco mais específico (creatininofosfoquinase porção MB – e com ele seus difíceis critérios: p.e.: relações CK total/MB). Finalmente, temos um marcador extremamente específico da injúriamiocárdica como a troponina. A evolução dos biomarcadores permitiu a simplificação dos protocolos de dor torácica, e a redução de alta inapropriada de pacientes com SCA.^2^ Devido à alta sensibilidade e especificidade da troponina, no quarto consenso sobre a definição universal de Infarto Miocárdico, se chegou à conclusão de que para estabelecer o diagnóstico clínico são necessários a elevação acima do percentil 99 deste biomarcador, associado a evidência clínica de isquemia miocárdica.^3^ Dado ao baixo corte da troponina, neste consenso há dúvidas em relação à relevância clínica.

Nesta edição dos Arquivos Brasileiros de Cardiologia, Tapas-Filho et al.,^4^ comparam o nível de corte percentil 99 versus o corte da bula do fabricante da troponina. Eles observaram que o valores utilizados de troponina elevados acima do percentil 99 pela 4^a^ Definição Universal de Infarto foram uteis em relação ao prognóstico, ou seja, foram capazes de prever o desfecho composto de óbito e reinfarto em até 30 dias. Uma observação adicional é que níveis de troponina minimamente elevados possibilitaram estratificar melhor os pacientes e identificar aqueles com maior probabilidade de se beneficiarem da estratégia invasiva precoce e de procedimentos de revascularização coronária.

Em relação ao trabalho publicado, apesar de dar suporte às recomendações, há algumas questões a se analisar. Primeiramente, trata-se de um registro de um único centro, com uma amostra limitada (494 pacientes), dentre os quais, os pacientes com troponina entre 0,034 e 0,12ng/dL foram somente 39. Segundo, observamos que a mortalidade dos grupos é baixa (2,4% a 3,9%) no registro, o que pode ser explicado pela população de baixo risco (GRACE SCORE: 102 (trop > 0,034-0,12ng/dL) x 120 (trop >0,12 ng/dL)). Outra possível explicação para a baixa mortalidade mencionada pelos autores é a alta taxa de estratégia invasiva e revascularização coronária precoce. Os níveis mais elevados de troponina apresentaram maior incidência de reinfarto (16,2% versus 4,8%), e ocorreram principalmente nos primeiros 15 dias. No estudo não ficaram claras as causas desse aumento. Podemos especular: revascularização incompleta? Infarto relacionado a procedimento (IAM tipo IV ou V)? São questões a serem analisadas com cuidado.

Além da limitação quanto ao tamanho da amostra do estudo, outro ponto de atenção é o período de seguimento. Quando comparamos com o registro SWEDEHEART (com mais de 48.000 pacientes incluídos), e a análise deste subgrupo (9.800 pacientes), acompanhado por dez anos, observou-se aumento dos eventos cardiovasculares nesta população, na ordem de 15,4%.^5^ Isto reforça a importância de pequenos aumentos da troponina como marcador de prognóstico a longo prazo.

Se por um lado, abaixar o ponto de corte dos biomarcadores é preditor de eventos, por outro lado há preocupação na redução da especificidade do teste, com aumento no número de falso positivos,^6^ o que poderia levar a procedimentos desnecessários, e ao aumento, por exemplo, de coronariografias sem lesões coronarianas (os chamados “cates brancos”). O que pode estigmatizar o paciente e expor a complicações relacionados à assistência. No registro de Tapas-Filho,^4^ observamos que em pacientes com níveis mais baixos de troponina, 92% foram submetidos a coronariografia e a taxa de revascularização foi de > 75% (semelhante ao grupo de troponina mais elevada). Mesmo assim, reforçamos que em geral 25% dos pacientes poderiam não ter sido submetidos a testes invasivos.

Sob nosso ponto de vista, o momento é de buscar marcadores, que evitem que pacientes sejam submetidos à estratégia invasiva desnecessariamente. Para termos a dimensão dos números, se considerarmos aproximadamente 110.000 revascularizações realizadas pelo sistema único de saúde (SUS) em 2019,^1^ estaríamos falando aproximadamente de 35.000 pacientes submetidos a coronariografia desnecessariamente por ano! Avançamos muito com esses novos “super” marcadores, melhoramos nosso diagnóstico e capacidade de prever eventos, mas é a hora de saber a melhor maneira de utilizá-los na prática clínica e reduzir procedimentos desnecessários.
