# Supplementary material for: Troponina – Use com Sabedoria. E como mais um Instrumento na Clínica
Source: Arq Bras Cardiol. 2022 Jun 6;118(6):1016–7. [Article in Portuguese] doi: 10.36660/abc.20220353 (PMC9345153; doi:10.36660/abc.20220353)
Supplement: Translation

# Translation: Troponin – Use it wisely. And as Another Instrument in the Clinic

**Authors:** Ricardo Wang, Estevāo Lanna Figueiredo

**Affiliations:** 1 Belo Horizonte MG Brazil Instituto Orizonti Hospital das Clínicas da Universidade Federal e Minhas Gerais, Belo Horizonte, MG – Brazil

**Keywords:** Myocardial Infarction, Biomarkers, Troponin, Mortality, Epidemiology, Myocardial Ischemia, Myocardial Revascularization, Acute Coronary Syndrome, Prognosis

Acute myocardial infarction (AMI) and stable chronic coronary disease are the main causes of mortality in Brazil.^1^ In 2019, it was responsible for more than 170,000 deaths in Brazil. Given its severity, the Cardiology made a great effort to constantly improve the tools for the correct diagnosis to avoid the release of patients with Acute Coronary Syndrome (ACS) and its clinical and legal consequences. They are considered pillars for the diagnosis and a good anamnesis with the characterization of the type of pain, electrocardiographic changes, and biomarkers (mainly troponin).

Biomarkers play an important role in recognizing ACS, and diagnostic algorithms have adapted as they evolve. At first, they were nonspecific markers (e.g., lactic dehydrogenase, oxacetic transaminase, total creatine phosphokinase – CK). Then they evolved to a slightly more specific marker (creatine phosphokinase MB portion) with its difficult criteria: e.g., total CK/MB). Finally, we have an extremely specific marker of myocardial injuries, such as troponin. The evolution of biomarkers has allowed the simplification of chest pain protocols and the reduction of inappropriate discharge of patients with ACS.^2^ Due to troponin’s high sensitivity and specificity, in the fourth consensus on the universal definition of myocardial infarction, it was concluded that to establish the clinical diagnosis, an elevation above the 99th percentile of this biomarker was associated with clinical evidence of myocardial infarction ischemia.^3^ Given the low cutoff for troponin, there are doubts in this consensus regarding the clinical relevance.

In this issue of the *Arquivos Brasileiros de Cardiologia*, Tapas-Filho et al.^4^ compare the 99th percentile cutoff level versus the troponin manufacturer’s label cutoff. They observed that the troponin values above the 99th percentile used by the 4^th^ Universal Definition of Infarction were useful in prognosis; they could predict the composite outcome of death and reinfarction within 30 days. An additional observation is that minimally elevated troponin levels made it possible to stratify patients better and identify those most likely to benefit from early invasive strategy and coronary revascularization procedures.

Despite supporting the recommendations, some issues are to be analyzed regarding the published work. First, it is a single-center registry with a limited sample (494 patients), among which patients with troponin between 0.034 and 0.12ng/dL were only 39. Second, we observed that the mortality of the groups is low (2.4% to 3.9%) in the registry, which can be explained by the low-risk population (GRACE SCORE: 102 (trop > 0.034-0.12ng/dL) x 120 (trop >0.12 ng/dL)). Another possible explanation for the low mortality mentioned by the authors is the high rate of invasive strategy and early coronary revascularization. Higher troponin levels had a higher incidence of reinfarction (16.2% versus 4.8%) and occurred mainly in the first 15 days. In the study, the causes of this increase were not clear. We can speculate: incomplete revascularization? Procedure-related infarction (type IV or V AMI)? These are issues to be carefully considered.

In addition to the limitation of the study sample size, another point of attention is the follow-up period. Compared with the SWEDEHEART registry (with more than 48,000 patients included) and the analysis of this subgroup (9,800 patients), followed for ten years, an increase in cardiovascular events was observed in this population in the order of 15.4%.^5^ This fact reinforces the importance of small increases in troponin as a long-term prognostic marker.

If, on the one hand, lowering the cutoff point of biomarkers is a predictor of events, on the other hand, there is concern about reducing the specificity of the test, with an increase in the number of false positives,^6^ which could lead to unnecessary procedures, and an increase, for example, coronary angiographies without coronary lesions (so-called “white catheters”), which can stigmatize the patient and expose them to complications related to care. In the Tapas-Filho^4^ registry, we observed that in patients with lower troponin levels, 92% underwent coronary angiography, and the revascularization rate was > 75% (similar to the higher troponin group). We emphasize that, in general, 25% of patients could not have undergone invasive tests.

From our point of view, the time is now to look for markers that prevent patients from being unnecessarily submitted to the invasive strategy. To have the dimension of the numbers, if we consider approximately 110,000 revascularizations performed by the Unified Health System (SUS) in 2019,^1^ we would be talking about approximately 35,000 patients undergoing coronary angiography unnecessarily per year! We have advanced a lot with these new “super” markers, we have improved our diagnosis and ability to predict events, but it is time to know the best way to use them in clinical practice and reduce unnecessary procedures.
